# Silencing long non-coding RNA MIAT ameliorates myocardial dysfunction induced by myocardial infarction *via* MIAT/miR-10a-5p/EGR2 axis

**DOI:** 10.18632/aging.202785

**Published:** 2021-03-26

**Authors:** Xiangke Cao, Qinghua Ma, Bin Wang, Qingqiang Qian, Ning Liu, Tiejun Liu, Xiaoliu Dong

**Affiliations:** 1 School of Life Sciences, North China University of Science and Technology, Tangshan 063210, P.R. China; 2 Department of Preventive Health, The Third People’s Hospital Of Xiangcheng District In Suzhou, Suzhou 215134, P.R. China; 3 Department of Pediatrics, North China University of Science and Technology Affiliated Hospital, Tangshan 063000, P.R. China; 4 Department of Neurology, Tangshan Gongren Hospital, Tangshan 063000, P.R. China; 5 Department of Cardiovascular Diseases, North China University of Science and Technology Affiliated Hospital, Tangshan 063000, P.R. China; 6 Department of Anesthesiology, North China University of Science and Technology Affiliated Hospital, Tangshan 063000, P.R. China; 7 Department of Neurology, Tangshan People’s Hospital, Tangshan 063001, P.R. China

**Keywords:** long non-coding RNA myocardial infarction-associated transcript, microRNA-10a-5p, early growth response gene-2, myocardial infarction, cardiac injury

## Abstract

Long non-coding RNA (lncRNA) myocardial infarction-associated transcript (MIAT) has been widely-demonstrated to function as diagnostic markers for acute myocardial infarction (MI). This study was designed to explore the modulatory role of MIAT and its underlying molecular mechanism in MI. Firstly, MI mouse model was developed *via* ligation of the descending branch of the left coronary artery, and cell model was established through exposure to hypoxic conditions*.* Online prediction indicated that MIAT could bind to microRNA-10a-5p (miR-10a-5p), while miR-10a-5p was highlighted to bind to early growth response gene-2 (EGR2). MIAT and EGR2 were subsequently determined to be highly-expressed, whereas miR-10a-5p was found to be poorly-expressed in cardiomyocytes exposed to hypoxia as well as in MI mice using RT-qPCR and Western blot assay. The binding relationships between MIAT and miR-10a-5p, and between miR-10a-5p and EGR2 were further confirmed by dual-luciferase reporter and RNA immunoprecipitation assays. The results of *in vitro* and *in vivo* experimentation also suggested that overexpression of miR-10a-5p or silencing of MIAT and EGR2 reduced cardiomyocyte apoptosis and increased ATP content, thus alleviating the impairment of cardiac function following MI. In a word, inhibition of MIAT protects against cardiac dysfunction induced by MI through the crosstalk with miR-10a-5p/EGR2.

## INTRODUCTION

Myocardial infarction (MI) is clinically differentiated into three types, which are as follows: type 1 is consequent of plaque rupture, type 2 is caused by oxygen supply/demand imbalance, and type 3 is characterized as MI with various cardiac biomarkers in the blood. Among them, myocardial injury is manifested with type 2 MI, as evidenced by the severely elevated cardiac troponin values [1]. The principal risk factors for MI include hypertension, hypercholesterolemia, diabetes, obesity, and smoking. Clinical outcome has witnessed significant improvement over the past two decades due to variations in patient populations, increased revascularization procedures application, and superior medications [2]. However, MI still persists as a primary cause of deaths worldwide, and the demand for alternative therapy is for urgency because the cardiac muscle cannot regenerate after severe injury [3]. Therefore, it is urgent to elucidate a new therapeutic target to alleviate subsequent myocardial injuries in MI.

Non-coding RNAs (ncRNAs), like microRNAs (miRs) and long ncRNAs (lncRNAs), vitally principally function in muscle regulation, and have been further demonstrated as viable targets for heart disease treatment [4]. LncRNAs have been indicated to serve as biomarkers of several cardiac ailments like cardiac hypertrophy and cardiac fibrosis [5–7]. Besides, some heart-specific lncRNAs such as Novlnc6 and Novlnc15 have been reported to be implicated in maladaptive remodelling, cardiac function and possibly cardiac regeneration post-MI [8]. As a non-coding functional RNA with five exons, myocardial infarction-associated transcript (MIAT), located at 22q12.1 with a length of 30,051 bp, is closely associated with MI manifestation [9, 10]. Intriguingly, this particular lncRNA has recently been speculated to function as a diagnostic marker for acute MI [11, 12]. The significance of myocardial MIAT has been identified in other heart diseases as well. A recent study elicited that MIAT is highly expressed in the angiotensin II-treated cardiomyocytes, and down-regulation of MIAT suppresses angiotensin II-induced cardiac hypertrophy by regulation of the miR-93/Toll-like receptor 4 axis [13]. MIAT expression was aberrantly elevated in cardiac diseases like cardiomyopathy and cardiac fibrosis [14, 15], suggesting the potential functionality of MIAT in cardiac disorders. However, the mechanism by which MIAT functions in MI has not been particularly identified yet. Existing studies have proposed that MIAT could competitively bind to miRNAs to up-regulate the gene expression [16, 17]. Additionally, lncRNA-miRNA interaction prediction by a combination of Starbase, miRcode, and DIANA-LncBase V2 prior to our study identified the presence of a specific binding relation between MIAT and miR-10a-5p. A poor miR-10a-5p expression is a tangible risk factor for hypertension [18], an acclaimed risk factor of MI. Also, a study has elucidated that miR-10a may impact the cardiac function after MI [19]. Additionally, early growth response gene-2 (EGR2) has been identified to be pro-apoptotic that can be repressed by miR-150, thus further protecting the cardiac muscle from detrimental ischemic injury [20]. Interestingly, bioinformatics analysis of our study also identified a potential binding relationship between miR-10a-5p and EGR2. Conjointly, from the aforementioned literature, it is hypothesized that the interaction among MIAT, EGR2, and miR-10a-5p may participate in the progression of MI. Therefore, our study was designed to investigate the regulatory role of MIAT in MI with the involvement of miR-10a-5p and EGR2.

## RESULTS

### MIAT was up-regulated in the cardiomyocytes exposed to hypoxia

**Figure 1:**
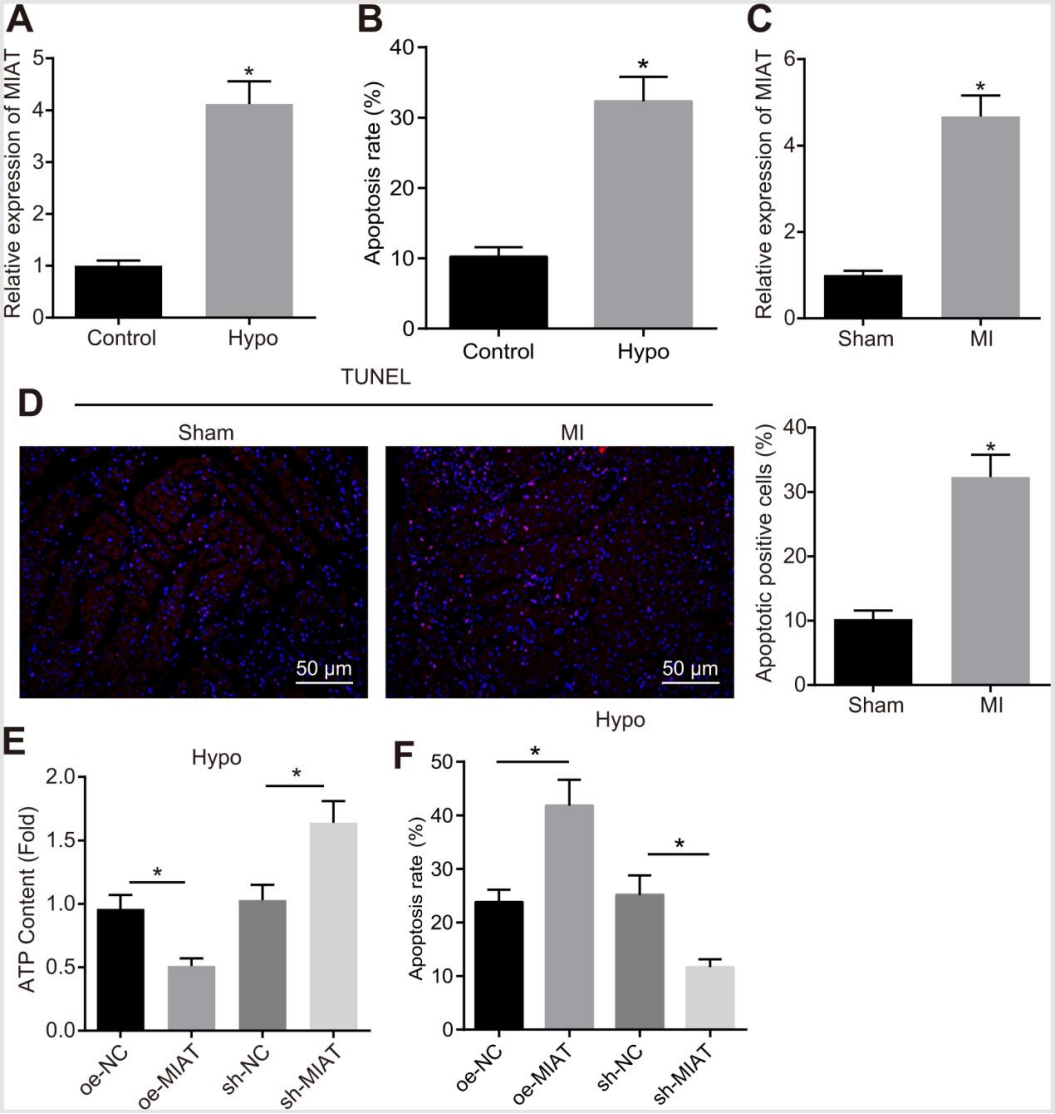
**Upregulation of MIAT in hypoxic cardiomyocytes and myocardial tissues from the MI mice.** (**A**) MIAT expression pattern in the cardiomyocytes determined by RT-qPCR normalized to GAPDH. (**B**) The cardiomyocyte apoptosis detected by flow cytometry. (**C**) RT-qPCR determination of MIAT expression pattern in the cardiomyocytes in mice 28 d after MI modeling (n = 10). (**D**) The apoptosis of cardiomyocytes in mice 28 d after MI modeling showed by TUNEL staining (scale bar = 50 μm). (**E**) The ATP content in hypoxic cardiomyocytes after alteration of MIAT. (**F**) The apoptosis of hypoxic cardiomyocytes after alteration of MIAT analyzed by flow cytometry. * *p* < 0.05. The above data were all measurement data, and expressed as mean ± standard deviation. The unpaired *t* test was used for comparison between two groups, and one-way ANOVA was applied for comparison between multiple groups followed by Tukey’s post hoc test. All the data was collected from 3 independent experiments respectively.

An abnormally high expression pattern of MIAT has been documented in various heart diseases [14, 15]. This study intends to further elucidate the regulatory role of MIAT in MI. First, an *in vitro* cardiomyocyte model of MI was established by exposure to hypoxia, where the MIAT expression pattern was determined by reverse transcription quantitative polymerase chain reaction (RT-qPCR), and the degree of apoptosis was assessed by flow cytometry. The results showed that the MIAT expression pattern (Figure 1A) and apoptosis (Figure 1B) were significantly up-regulated in the HL-1 cells after 24 h of hypoxia treatment. In the animal experiment, after 28 days of MI establishment, the cell apoptosis and MIAT expression pattern in the myocardial tissues were assessed. The results showed that the MIAT expression pattern (Figure 1C) and cell apoptosis (Figure 1D) were significantly increased in mice with MI compared with the sham-operated mice (*p* < 0.05). To further investigate the effects of MIAT on cardiomyocytes, we overexpressed or knocked down MIAT in the hypoxic cardiomyocytes to evaluate the effect of MIAT at the cellular level. Acknowledging that the normal function of cardiomyocytes is dependent on mitochondria-associated energy metabolism [21], the measurement of cellular ATP was conducted in an attempt to assess the function of cardiomyocytes. As shown in Figure 1E, 1F, the ATP content of the hypoxic cardiomyocytes was significantly lowered and the apoptosis of hypoxic cardiomyocytes was increased by overexpressing MIAT, both of which were contrasting in the hypoxic cardiomyocytes transfected with sh-MIAT (Figure 1G, *p* < 0.05). Therefore, our findings revealed that the MIAT expression pattern was significantly increased in cardiomyocytes under hypoxic condition and the knockdown of MIAT reduced the injury to cardiomyocytes caused by hypoxia treatment.

### MIAT promoted the apoptosis of cardiomyocytes by decreasing miR-10a-5p expression

**Figure 2:**
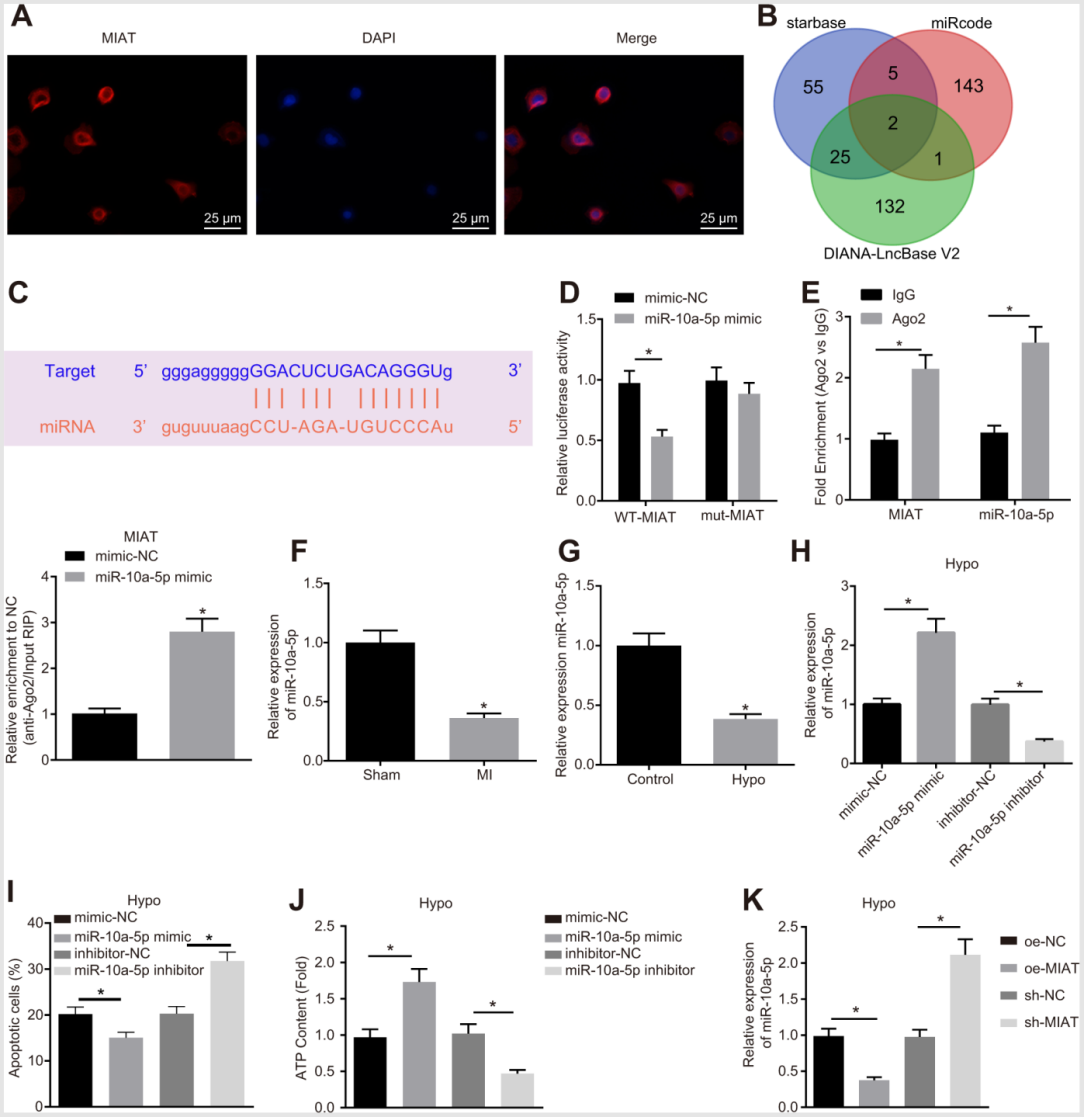
**MIAT downregulated miR-10a-5p and suppressed cardiomyocyte apoptosis.** (**A**) The location of MIAT in cardiomyocytes assessed by FISH assay (400 ×). (**B**) The downstream miRNAs of MIAT predicted by Starbase, miRcode, and DIANA-LncBase V2. (**C**) The predicted binding sites between MIAT and miR-10a-5p. (**D**) The interaction between MIAT and miR-10a-5p detected by dual-luciferase reporter assay. (**E**) Binding of MIAT to miR-10a-5p relative to IgG confirmed by RIP assay. (**F**) miR-10a-5p expression normalized to U6 in the myocardial tissues of mice assessed by RT-qPCR (n = 10). (**G**) miR-10a-5p expression normalized to U6 in the hypoxic cardiomyocytes assessed by RT-qPCR. (**H**) miR-10a-5p expression normalized to U6 in the hypoxic cardiomyocytes after transfection with miR-10a-5p mimic or miR-10a-5p inhibitor assessed by RT-qPCR. (**I**) Apoptosis of hypoxic cardiomyocyte after transfection with miR-10a-5p mimic or miR-10a-5p inhibitor analyzed by flow cytometry. (**J**) The content of ATP in the hypoxic cardiomyocytes after transfection with miR-10a-5p mimic or miR-10a-5p inhibitor. (**K**) miR-10a-5p expression normalized to U6 in HL-1 cells under hypoxic conditions after alteration of MIAT determined by RT-qPCR. * *p* < 0.05. The above data were all measurement data, and expressed as mean ± standard deviation. The unpaired *t* test was used for comparison between two groups, and one-way ANOVA was applied for comparison between multiple groups followed by Tukey’s post hoc test. All the data was collected from 3 independent experiments respectively.

To further study the regulatory mechanism of MIAT, fluorescence *in situ* hybridization (FISH) was conducted with the results demonstrated that MIAT was predominantly localized in the cytoplasm (Figure 2A). Existing reports have illustrated that MIAT can competitively bind to miRNA [16, 17]. In our study, a combination of the Starbase, miRcode, and DIANA-LncBase V2 databases were employed to predict the downstream miRNAs of MIAT, which revealed two intersection miRNAs, miR-10a-5p and miR-10a-5p, among the miRNAs obtained from the three databases (Figure 2B). Prior research proposed that miR-10a might be of significant functionality in the regulation of cardiac function after MI [19]. Specific binding sites between MIAT and miR-10a-5p were obtained (Figure 2C). To validate this result, dual-luciferase reporter assay was performed. The results revealed that in the normal cardiomyocytes, the luciferase activity of the cells co-transfected with miR-10a-5p mimic and wild type (WT)-MIAT significantly decreased (*p* < 0.05) but no significant difference was observed in the luciferase activity of the cells co-transfected with mutant (MUT)-MIAT, indicating the ability of MIAT to specifically bind to miR-10a-5p (Figure 2D). In addition, the RNA immunoprecipitation (RIP) experiments revealed that the MIAT and miR-10a-5p enrichment in the cells incubated with anti-Ago2 significantly increased compared to the cells incubated with anti-IgG (Figure 2E, *p* < 0.05), indicating that MIAT could interact with miR-10a-5p, thus further forming a RNA-induced silencing complex. Hence, with the preceding results, we speculated that MIAT might impair the cardiomyocyte function by interacting with miR-10a-5p. To further investigate the effect of MIAT on miR-10a-5p expression pattern, the miR-10a-5p expression pattern was determined in the myocardial tissues of MI mice and hypoxic cardiomyocytes (Figure 2F). In comparison with the mice subjected to the sham operation, the miR-10a-5p expression pattern in the MI mice was significantly down-regulated (*p* < 0.05). In addition, the miR-10a-5p expression pattern in the cardiomyocytes decreased significantly after hypoxia treatment (Figure 2G). Then, the miR-10a-5p mimic and miR-10a-5p inhibitor were introduced into the hypoxic cardiomyocytes (Figure 2H) for further investigation on the effects of miR-10a-5p. The results uncovered that after transfection with the miR-10a-5p mimic, the apoptosis of hypoxic cardiomyocyte was diminished and the ATP synthesis was increased, while miR-10a-5p inhibitor transfection led to increased apoptosis and reduced ATP content (Figure 2I, 2J). Then, the effect of MIAT on the miR-10a-5p expression decreased was further studied in the hypoxic cardiomyocytes. Overexpression of MIAT reduced the miR-10a-5p expression pattern while silencing of MIAT increased the miR-10a-5p expression pattern (Figure 2K). Based on the above results, it can be conferred that MIAT induced the apoptosis of cardiomyocytes by downregulating miR-10a-5p.

### miR-10a-5p suppressed the apoptosis of cardiomyocytes *via* regulating EGR2 negatively

**Figure 3:**
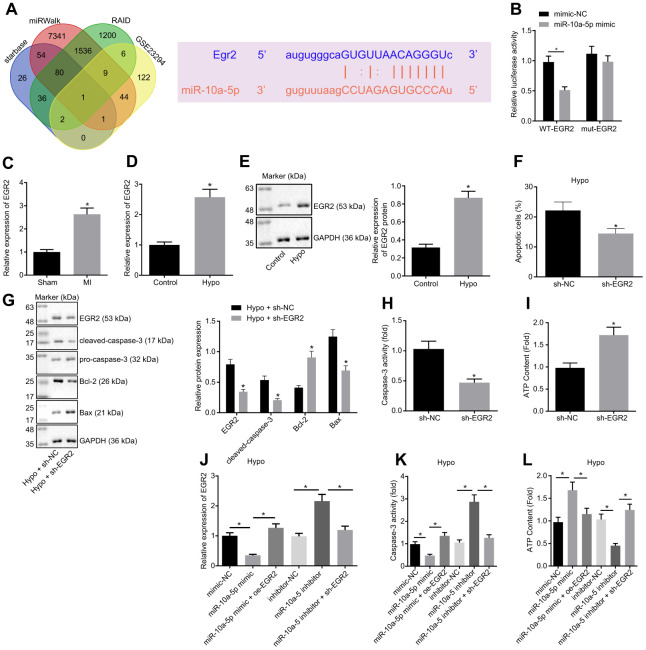
**Overexpression of miR-10a-5p decreased cardiomyocyte apoptosis through inhibiting EGR2.** (**A**) The target genes of miR-10a-5p predicted using Starbase, miRWalk, and RAID databases and the up-regulated genes in MI-related dataset GSE23294 and the potential binding sites of miR-10a-5p on EGR2. (**B**) The luciferase activity measured by dual-luciferase reporter gene assay. (**C**) Expression of EGR2 in myocardial tissues normalized to GAPDH determined by RT-qPCR (n = 10). (**D**) The expression pattern of EGR2 in hypoxic cardiomyocytes normalized to GAPDH determined by RT-qPCR. (**E**) The expression pattern of EGR2 in cardiomyocytes normalized to GAPDH determined by Western blot analysis. (**F**), Apoptosis of cardiomyocytes after EGR2 silencing determined by flow cytometry. (**G**) The expression patterns of EGR2, cleaved-caspase-3, Bax and Bcl-2 in cardiomyocytes after EGR2 silencing normalized to GAPDH determined by Western blot analysis. (**H**) The caspase-3 activity in hypoxic cardiomyocytes after EGR2 silencing. (**I**) The ATP content in hypoxic cardiomyocytes after EGR2 silencing. (**J**) The expression pattern of EGR2 in the hypoxic cardiomyocytes after alteration of EGR2 and/or miR-10a-5p normalized to GAPDH determined by RT-qPCR. (**K**) The caspase-3 activity in hypoxic cardiomyocytes after alteration of EGR2 and/or miR-10a-5p determined by RT-qPCR. (**L**) The ATP content in hypoxic cardiomyocytes after alteration of EGR2 and/or miR-10a-5p. * *p* < 0.05. The above data were all measurement data, and expressed as mean ± standard deviation. The unpaired *t* test was adopted for comparison between two groups. One-way ANOVA was adopted for comparison among multiple groups with Tukey’s post hoc test. All data was generated from 3 independent experiments respectively.

To further investigate the regulatory mechanism of miR-10a-5p, the target genes of miR-10a-5p were predicted through RAID, Starbase, and miRcode databases in combination with the results of upregulated genes from the MI-related microarray data GSE23294. Our findings revealed that EGR2 could bind to miR-10a-5p with the corresponding binding region predicted by the online website (Figure 3A). Furthermore, dual-luciferase reporter gene assay performed in the HEK293T cells showed that the luciferase activity of the 3'UTR in WT-EGR2 was significantly lowered by miR-10a-5p mimic (Figure 3B, *p* < 0.05), while the luciferase activity of 3'UTR in MUT-EGR2 showed no significant differences (*p* > 0.05). The aforementioned results indicated that miR-10a-5p could bind to specifically EGR2.

The effect of EGR2 on cardiomyocyte function was further explored. EGR2 expression pattern in the myocardial tissues from MI mice and hypoxic cardiomyocytes was determined (Figure 3C). The results showed that the EGR2 expression pattern was significantly up-regulated in MI mice compared to the sham-operated mice (*p* < 0.05). The EGR2 expression pattern in the cardiomyocytes significantly increased after hypoxia treatment (Figure 3D, 3E). Furthermore, EGR2 silencing reduced the apoptosis of cardiomyocytes under hypoxic conditions (Figure 3F). It has been reported that the downstream of EGR2 included numerous effector molecules involved in apoptosis, such as caspase-3 [22]. Therefore, we assessed the expression patterns of pro-caspase-3, cleaved-caspase-3, Bax and Bcl-2, and the results showed that under exposure to hypoxia, the expression patterns of cleaved-caspase-3, Bax, caspase-3 activity and ATP content decreased significantly while the Bcl-2 expression increased in the cells transfected with sh-EGR2 (Figure 3G–3I). These findings suggested that silencing EGR2 suppressed cardiomyocyte apoptosis.

To demonstrate that miR-10a-5p functioned by regulating EGR2, the miR-10a-5p and/or EGR2 were overexpressed or silenced in the hypoxic cardiomyocytes. The results suggested that in the hypoxic model, the EGR2 expression pattern (Figure 3J) and caspase-3 activity (Figure 3K) significantly decreased, while the ATP synthesis increased (Figure 3L) in the cells transfected with the miR-10a-5p mimic, opposite to the changes observed in the presence of miR-10a-5p inhibitor (Figure 3J–3L). In addition, the EGR2 expression pattern (Figure 3J) and caspase-3 activity (Figure 3K) were significantly elevated in the cells co-transfected with miR-10a-5p mimic and oe-EGR2 compared to the cells transfected with the miR-10a-5p mimic alone, accompanied by decreased ATP biosynthesis (Figure 3L). In comparison with the transfection of miR-10a-5p inhibitor alone, co-transfection of miR-10a-5p inhibitor and sh-EGR2 led to significant reduction of the EGR2 expression pattern (Figure 3J) and caspase-3 activity (Figure 3K), with an increase in the ATP content in the cardiomyocytes (Figure 3L). In summary, miR-10a-5p participated in the protection of cardiomyocytes under hypoxic condition by negatively regulating EGR2.

### MIAT promoted EGR2 expression by suppressing miR-10a-5p, and increased apoptosis of cardiomyocytes under hypoxia condition

**Figure 4:**
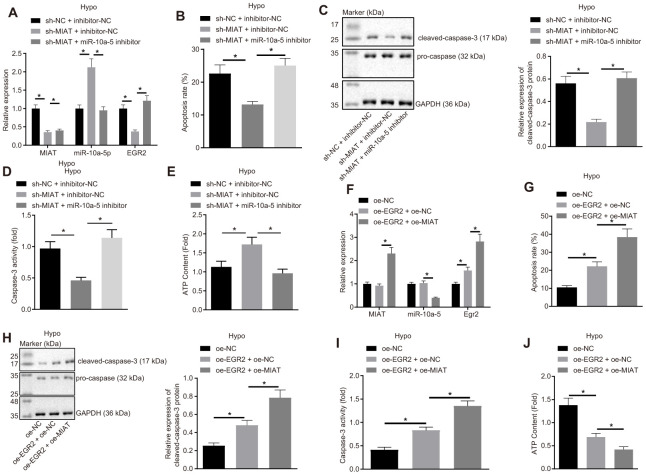
**MIAT competitively binds to miR-10a-5p to upregulate EGR2 and further increase apoptosis of cardiomyocytes under exposure to hypoxia.** (**A**) The expression of MIAT, miR-10a-5p and EGR2 in cardiomyocytes after inhibition of MIAT and/or miR-10a-5p determined by RT-qPCR. (**B**) Apoptosis of cardiomyocytes after inhibition of MIAT and/or miR-10a-5p detected by flow cytometry. (**C**) Western blot showing the expression patterns of cleaved-caspase-3 and caspase-3 in hypoxic cardiomyocytes after inhibition of MIAT and/or miR-10a-5p. The expression level is normalized to GAPDH. (**D**) The caspase-3 activity of cardiomyocytes under hypoxic conditions after inhibition of MIAT and/or miR-10a-5p. (**E**) The ATP content in cardiomyocytes after inhibition of MIAT and/or miR-10a-5p under hypoxic conditions. (**F**) The expression patterns of MIAT, miR-10a-5p and EGR2 in cardiomyocytes after MIAT and/or EGR2 overexpression determined by RT-qPCR normalized to GAPDH and U6. (**G**) The apoptosis of cardiomyocytes under hypoxic conditions after MIAT and/or EGR2 overexpression detected by flow cytometry. (**H**) The expression of cleaved-caspase-3 and pro-caspase-3 in the hypoxic cardiomyocytes after MIAT and/or EGR2 overexpression normalized to GAPDH determined by Western blot analysis. (**I**) The caspase-3 activity of cardiomyocytes under hypoxic conditions after MIAT and/or EGR2 overexpression. (**J**) The ATP content in cardiomyocytes under hypoxic conditions in response to MIAT and EGR2 overexpression relative to ATP content after EGR2 overexpression alone (fold). * *p* < 0.05, the above data were all measurement data, and expressed as mean ± standard deviation. The data comparison among multiple groups was performed using one-way ANOVA and Tukey’s post hoc test. The experiment was repeated 3 times independently.

Our results demonstrated the interaction between MIAT/miR-10a-5p and miR-10a-5p/EGR2. It was speculated that MIAT might function as a regulator of EGR2-mediated apoptosis by competitively binding to miR-10a-5p. To further validate this hypothesis, the expression patterns of MIAT, miR-10a-5p and EGR2 were determined after inhibition of MIAT and miR-10a-5p in the cardiomyocytes under hypoxic conditions (Supplementary Figure 1A). In comparison with the silencing of MIAT alone, silencing both miR-10a-5p and MIAT simultaneously increased the EGR2 expression pattern in the cardiomyocytes under hypoxic conditions (Figure 4A), promoted apoptosis (Figure 4B), cleaved caspase-3 expression pattern (Figure 4C) and caspase-3 activity (Figure 4D), and decreased the ATP content (Figure 4E) (all *p* < 0.05). Therefore, MIAT increased the EGR2 expression pattern to subsequently induce apoptosis by reducing miR-10a-5p in *in vitro* hypoxia model.

Furthermore, in order to ascertain whether EGR2 is an effector protein participating in the MIAT/miR-10a-5p axis, MIAT and/or EGR2 were overexpressed in the cell model (Supplementary Figure 1B). The results exhibited that compared with the cells co-transfected with oe-EGR2 and oe-NC, cells co-transfected with oe-MIAT and oe-EGR2 under hypoxic conditions exhibited reduced miR-10a-5p expression pattern, increased MIAT and EGR2 expression patterns (Figure 4F), apoptosis (Figure 4G), cleaved capsase-3 expression pattern (Figure 4H) and caspase-3 activity (Figure 4I), along with reduced ATP synthesis (Figure 4J) (all *p* < 0.05). Collectively, MIAT facilitated the apoptosis of hypoxic cardiomyocytes by upregulating EGR2 *via* miR-10a-5p.

### Inhibition of MIAT improved cardiomyocyte function in mice after MI *via* miR-10a-5p-targeted EGR2

**Figure 5:**
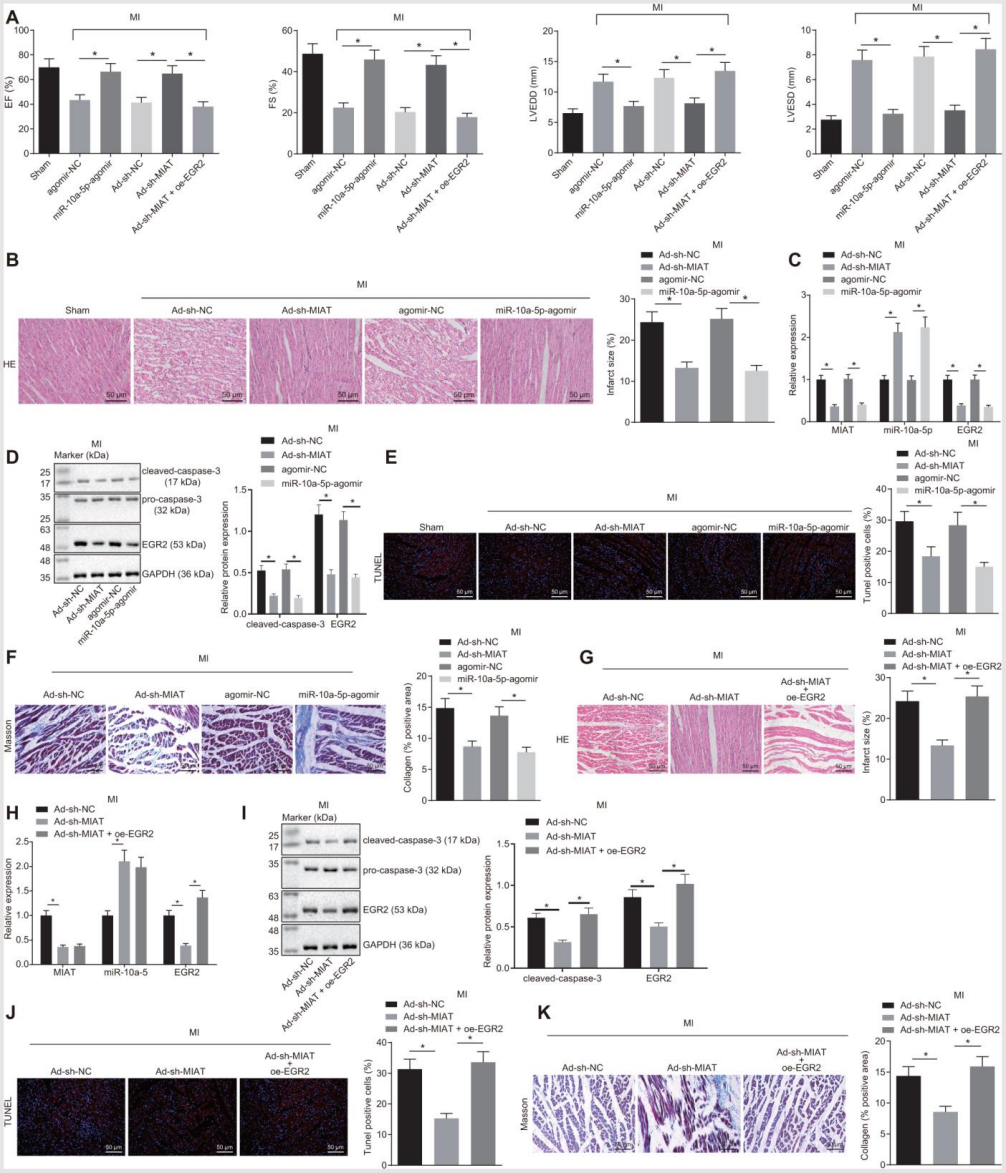
**MIAT silencing improves cardiomyocyte function in MI mice by inhibiting EGR2 through miR-10a-5p upregulation.** (**A**) EF, FS, LVEDD, and LVESD values in mice detected by small animal echocardiogram after MI modeling with alteration of MIAT, miR-10a-5p or EGR2. (**B**) Myocardial infarct size identified by HE staining after alteration of MIAT and miR-10a-5p (scale bar = 50 μm). (**C**) The expression patterns of MIAT, miR-10a-5p and EGR2 in the cardiac tissues of MI mice after alteration of MIAT and miR-10a-5p determined by RT-qPCR. (**D**) The expression patterns of cleaved caspase-3 and EGR2 normalized to GAPDH in the myocardial tissues of MI mice after alteration of MIAT and miR-10a-5p determined by Western blot analysis. (**E**) The apoptosis in myocardial tissues after alteration of MIAT and miR-10a-5p showed by TUNEL staining (scale bar = 50 μm). The red signal represented TUNEL-positive cells. (**F**) The collagen deposition in the myocardial tissues of MI mice after alteration of MIAT and miR-10a-5p showed by Masson staining (scale bar = 50 μm). The blue color represented collagen. (**G**) The myocardial infarct size in mice after alteration of MIAT and EGR2 showed by HE staining (scale bar = 50 μm). (**H**) The expression patterns of MIAT, miR-10a-5p and EGR2 in cardiac tissues of MI mice after alteration of MIAT and EGR2 determined by RT-qPCR. (**I**) Western blot showing the expression patterns of cleaved caspase-3, pro-caspase-3 and EGR2 in mouse myocardial tissues after alteration of MIAT and EGR2. The expression level is normalized to GAPDH. (**J**) The apoptosis in myocardial tissues of MI mice after alteration of MIAT and EGR2 determined by TUNEL staining (scale bar = 50 μm). The red signal represented TUNEL-positive cells. (**K**) The collagen deposition in the myocardial tissues of MI mice after alteration of MIAT and EGR2 showed by Masson staining (scale bar = 50 μm), and blue referred to collagen. The animals were selected blindly, and all values were measured on the same animal for 6 consecutive cardiac cycles. * *p* < 0.05. The above data were all measurement data, and expressed as mean ± standard deviation. The data of multiple groups were compared by one-way ANOVA and Tukey’s post hoc test. n = 10.

A mouse MI model was established to validate the role of the MIAT/miR-10a-5p/EGR2 axis *in vivo*. In light of the relevant studies that suggested changes in the expression patterns of MIAT and EGR2 after MI [13, 20], the mouse serum and myocardial tissue samples were collected after 28 days to explore the effect of MIAT and EGR2 on MI *in vivo*. Prior to the MI modeling, the mice were pre-injected with Ad-sh-MIAT, miR-10a-5p-agomir, Ad-oe-EGR2, or their negative controls *via* the tail vein. Cardiac function was assessed using animal echocardiography (Figure 5A). Left ventricular end-diastolic diameter (LVEDD), left ventricular end-systolic diameter (LVESD), left ventricular short-axis ejection fraction (EF), and shortening score (FS) were reflected by two-dimensional and M-mode echocardiography to evaluate the movement of the ventricular wall and functioning of the left ventricle. The results demonstrated that LVEDD and LVESD of the mice treated with miR-10a-5p-agomir were significantly lowered while the EF and FS were significantly elevated (Figure 5A). This indicated that up-regulation of miR-10a-5p improved cardiac function after MI. Instead, compared with the mice injected with the Ad-sh-MIAT, LVEDD and LVESD values of the mice injected with Ad-sh-MIAT + oe-EGR2 increased significantly, and EF and FS values decreased significantly (*p* < 0.05, Figure 5A), indicating that overexpression of EGR2 aggravated the cardiac dysfunction of mice after MI. In addition, hematoxylin-eosin (HE) staining showed infarcted myocardial tissues in MI mice (Figure 5B). The myocardial infarct size decreased in mice injected with Ad-sh-MIAT or miR-10a-5p-agomir, indicative of MI symptom alleviation. The expression patterns of MIAT, miR-10a-5p and EGR2 and the apoptosis indicators (Figure 5C–5E) were also determined. Our results presented that in mice injected with Ad-sh-MIAT or miR-10a-5p-agomir, MIAT, EGR2 and cleaved caspase-3 expression patterns and the number of apoptotic cells were significantly decreased, while the miR-10a-5p expression pattern increased. Masson staining showed that either silence of MIAT or overexpression of miR-10a-5p reduced the collagen deposition (Figure 5F). Additionally, in order to verify that EGR2 is an effector molecule of the MIAT/miR-10a-5p axis, the mice were injected with Ad-sh-MIAT and Ad-oe-EGR2. In comparison with the Ad-sh-MIAT injection group, the myocardial infarct size was significantly increased when the mice were injected with both Ad-sh-MIAT and oe-EGR2 (Figure 5G). The treatment with Ad-sh-MIAT and Ad-oe-EGR2 resulted in increased expression patterns of EGR2 (Figure 5H) and cleaved-caspase-3 (Figure 5I), and enhanced cardiomyocyte apoptosis (Figure 5J) and collagen deposition (Figure 5K) relative to the treatment with Ad-sh-MIAT alone. The aforementioned results suggested that silencing of MIAT improved cardiac function in mice after MI by downregulating miR-10a-5p-targeted EGR2.

## DISCUSSION

MI, as a common cause of mortality across the world, is the clinically defined as the progression of myocardial injury with detrimental symptoms of myocardial ischemia, increased cardiac troponin levels and insufficient oxygen supply [1, 23]. The poor outcome of patients with MI is attributable to the significant loss of cardiomyocytes and its impairment in the regeneration ability after MI [24]. Accumulating evidences highlights the vitality of lncRNAs in several cardiac diseases and with therapeutic capacity to treat cardiac injury [25, 26]. However, detailed studies regarding lncRNAs and its regulatory mechanism in MI are still insufficient. The current study aimed at ascertaining the role of lncRNA MIAT in MI. Our dissertation summarized that the lncRNA MIAT repression reduced the cardiomyocyte apoptosis by down-regulating miR-10a-5p-mediated EGR2, thus alleviating the subsequent myocardial injuries after MI.

We firstly identified the up-regulation of MIAT in the hypoxic cardiomyocytes and myocardial tissues of MI mice, where the ectopic expression of MIAT could intensify the apoptosis of hypoxic cardiomyocytes. In consistency with our finding, lncRNA X-inactive specific transcript (XIST) has an up-regulated expression in MI, which coherently elevates the degree of cardiomyocyte apoptosis as well [27]. Similarly, the lncRNA maternally expressed gene 3 (MEG3) is elevated under hypoxic condition and its knockdown in cardiomyocytes presents as a viable target therapy for MI treatment [23]. More recently, the lncRNA growth arrest-specific transcript 5 (GAS5) could evidently induce the apoptosis of cardiomyocytes under hypoxic conditions [28]. In our study, MIAT could inhibit the expression pattern of miR-10a-5p. According to a recent study, MIAT can fundamentally suppress miR-24 in cardiac fibroblasts as a pro-fibrotic lncRNA in the heart [15]. Interestingly, an elevated expression pattern of MIAT has been reported in cardiac hypertrophy in correlation with poor oncologic outcome by decreasing miR-93 [13]. Hence, from the preceding literature, it can be inferred that the up-regulation of MIAT in MI potentially plays a stimulative role in relation to the apoptosis of cardiomyocytes after MI *via* interaction with miR-10a-5p.

This study further revealed a poorly expressed miR-10a-5p pattern along with a highly expressed EGR2 pattern in MI and ascertained a relationship elucidating that overexpressed miR-10a-5p inhibited the expression of EGR2 and thus further reduced the apoptosis of cardiomyocytes. An existing study reported that miR-10a induces the formation of blood vessels in ischemic mice through activating Akt and stimulating the expression pattern of several angiogenic factors [19]. Similarly, the down-regulation of miR-10a in a mouse model of cardiac hypertrophy, elicited the potential of miR-10a as a promising target for cardiac hypertrophy management [29]. EGR2 has been proved to be pro-apoptotic and can be repressed by miR-150 in the cardiomyocytes, thus exercising cardioprotective properties during ischemic injury [20]. Meanwhile, our findings demonstrated that after EGR2 was silenced or miR-10a-5p was up-regulated, cleaved caspase-3 and Bax expression, caspase-3 activity and ATP content all decreased evidently, while the Bcl-2 expression pattern increased. As a vital component of metabolism, ATP is implicated in the systematic functioning of cardiomyocytes [21, 30]. Besides, ATP postconditioning has been investigated to serve as a cardioprotective approach to treat myocardial ischemia by preserving the overall cardiac function [31]. Bax, as a Bcl-2 family protein, is the central mediator of apoptosis [32]. The down-regulation of Bax and up-regulation of Bcl-2 induced by miR-208b overexpression are associated with the suppression of apoptosis under hypoxic conditions in MI [33]. Caspase-3 is an apoptotic signaling pathway-related protein that extensively participates in the induction of pyroptosis, a form of cell death [34, 35]. Another study revealed that attenuated cardiomyocyte apoptosis was accompanied by a decreased cleaved caspase-3 expression, which consequently conferred the protection against cardiac injury [36]. Therefore, these changes of the apoptotic mediators in the downstream of EGR2 further verified the pro-apoptotic properties of EGR2 in cardiomyocytes after MI. Furthermore, another study also elucidated that EGR2 promoted apoptosis in multiple cells by activating caspase-3 [22]. Our *in vivo* data mainly supported the conclusion that MIAT silencing or enhancement of miR-10a-5p contributed to a cardioprotective role in MI which was achieved by silencing EGR2.

**Figure 6:**
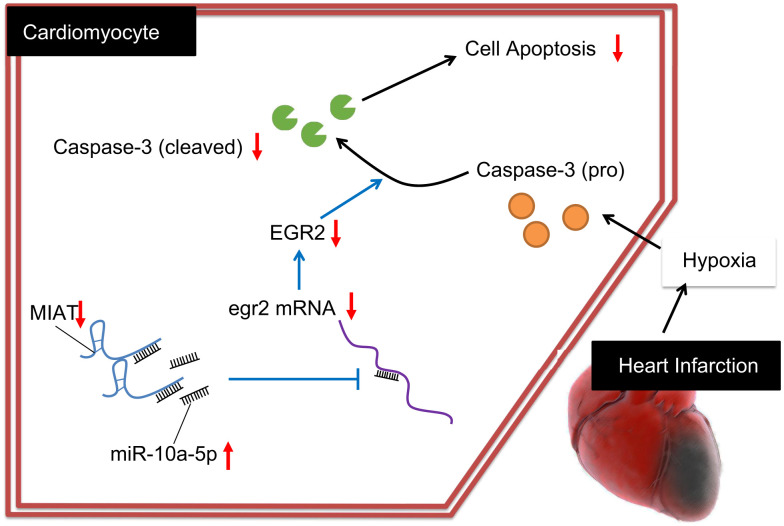
**Regulatory mechanism of MIAT in MI mice with the involvement of miR-10a-5p and EGR2.** Inhibition of the MIAT induces miR-10a-5p-dependent inhibition of EGR2 and reduces the production of cleaved caspase-3, ultimately suppressing the apoptosis of cardiomyocytes.

Collectively, our data exhibited MIAT up-regulation in MI. Moreover, MIAT silencing conferred a protective role against cardiomyocyte apoptosis *via* miR-10a-5p-dependent inhibition of EGR2 (Figure 6). Hence, from the aforementioned literature, it can be elucidated that the inhibition of MIAT could serve as a promising therapeutic method for treating MI by alleviating the myocardial injuries. However, the interaction of miRNA and lncRNA is an intriguing and significant research topic, and the underlying rules warrant further investigation for effective therapeutic modalities of cardiac diseases.

## MATERIALS AND METHODS

### Ethics statement

Experimental procedures in this study were performed with approval of the Tangshan People’s Hospital (ethical number: 20190815), and all animal experiments were conducted in strict accordance with the recommendations in the Guide for the Care and Use of Laboratory Animals of the National Institutes of Health. Adequate measures were taken to minimize the suffering of animals in this study.

### *In silico* analysis

Starbase (http://starbase.sysu.edu.cn/index.php), miRcode (http://www.mircode.org/), and DIANA-LncBase V2 (http://www.rna-society.org/raid/) databases were adopted to predict the downstream regulatory miRNAs of MIAT. Likewise, the Starbase, miRWalk (http://mirwalk.umm.uni-heidelberg.de/), and RAID (http://www.rna-society.org/raid/) databases were employed to predict the downstream targets of miR-10a-5p. Afterwards, MI-related microarray dataset (GSE23294) was retrieved from the Gene Expression Omnibus database. Using the “limma” package of the R language, the differentially expressed genes (DEGs) in 10 MI samples and 10 control samples from this dataset were screened with |logFC| > 1, *p* value < 0.01 as the criteria. Finally, the intersection genes between the DEG candidates and the up-regulated genes in another MI-related dataset GSE23294 were visualized using the Venn website (http://bioinformatics.psb.ugent.be/webtools/Venn/).

### Generation of MI mouse model

Seventy specific pathogen free (SPF) male C57BL/6J mice (age: 8 - 11 weeks, weight: 20.22 ± 1.46 g) were acquired from Beijing Vital River Laboratory Animal Technology Co., Ltd. (Beijing, China), among which 10 were grouped as the normal control, and the remaining 60 were reserved for the establishment of MI mouse models with a success rate of 83.33% (50/60). All mice were housed in an SPF animal laboratory on a normal diet with relative humidity of 60% - 65% and temperature at 22° C - 25° C.

Then the adenoviral vectors (1 × 10^9^ pfu/mouse, Shanghai GenePharma Co., Ltd., Shanghai, China) harboring sh-NC, sh-MIAT, oe-EGR2, agomir-NC, or miR-10a-5p agomir (2.5 μg/g, Ribobio Co., Ltd., Guangzhou, Guangdong, China) was injected into the C57BL/6 mice of the experimental group *via* the tail vein. These mice were fasted overnight before MI induction. Mice were anesthetized using an intraperitoneal injection with pentobarbital sodium (0.05 mg/g, P3761, Sigma-Aldrich, St. Louis, MO, USA). Next, the thoracic cavity was exposed and the left coronary artery descending branch (2 mm at the lower border of the left atrium junction) was ligated using a 7-0 surgical suture to establish a mouse model of MI. The 10 mice from the control group underwent the aforementioned surgical procedures without ligation. After surgery, the thoracic cavity was sutured and the condition and wound infection of the mice were observed. Samples were collected 28 days after surgery from the animals under anesthesia (method as described above), the eyeballs were harvested with a pair of curved tweezers, and 0.8 - 1.5 mL of blood was withdrawn using an anticoagulated blood collection tube (367947, BD bioscience, San Jose, CA, USA), followed by isolation of the heart.

### Assessment of cardiac function

Small animal echocardiography, the Vevo 2100 system (VisualSonics, Toronto, Canada) was introduced to measure LVEDD, LVESD, EF, and FS. The statistical formula was as follows: EF (%) = [(EDD3 − ESD3)/EDD3] × 100%; FS (%) = [(LVEDD−LVESD)/LVEDD] × 100%. All values were measured on the same animal selected randomly for 6 consecutive cardiac cycles.

### Culture of cardiomyocytes and establishment of a hypoxic cell model

The mouse cardiomyocyte cell line HL-1 cells were cultured using the Dulbecco’s modified Eagle’s medium (DMEM, Gibco™, Thermo Fisher, Waltham, MA, USA) containing a combination of 10% fetal bovine serum (FBS, Excell Bio, Genetimes, Shanghai, China), 100 U/mL penicillin and 100 μg/mL streptomycin in an incubator at 37° C with 5% CO_2_ and under saturated humidity.

The cells were transfected with sh-MIAT, oe-MIAT (pcDNA3.0-MIAT), oe-EGR2, miR-10a-5p mimic, miR-10a-5p inhibitor, sh-EGR2 or their negative controls (sh-NC, oe-NC [pcDNA3.0], mimic-NC and inhibitor-NC). The aforementioned shRNA sequences are shown in Table 1. The cells at passage 3 were seeded in a 6-well plate and cultured for 24 h. The cells were transfected upon 70% confluence according to the provided instructions of lipofectamine 2000 (11668-019, Invitrogen™, Carlsbad, CA, USA). After 6-8 h, the medium was renewed with fresh DMEM containing 10% FBS for subsequent experimentation after 24-48 h culture.

**Table 1:** shRNA sequences.

	**Sequences**
sh-NC	5'-CACCGTTCTCCGAACGTGTCACGTTTCAGAGAACGTGACACGTTCGGAGAATTTTTTG-3'
	5'-GATCCAAAAAATTCTCCGAACGTGTCACGTTCTCTTGAAACGTGACACGTTCGAGAA C-3'
sh-MIAT	5'-CCGGGTCCACCAGGTTAGCAATTAACTCGAGTTAATTGCTAACCTGGTGGACTTTTTG-3'
	5'-AATTCAAAAAGTCCACCAGGTTAGCAATTAACTCGAGTTAATTGCTAACCTGGTGGAC-3'
sh-EGR2	5'-CCGGGGGATCCGCATTTGCATAATTCTCGAGAATTATGCAAATGCGGATCCCTTTTTG-3'
	5'-AATTCAAAAAGGGATCCGCATTTGCATAATTCTCGAGAATTATGCAAATGCGGATCCC-3'

Note: sh/shRNA, short hairpin RNA; NC, negative control; MIAT, myocardial infarction-associated transcript; EGR2, early growth response gene-2.

As for the adenovirus infection, a section of 4 × 10^5^ of the cardiomyocytes were seeded in a 6-well plate after virus infection, and cultured using DMEM containing 10% FBS for 24 h, followed by the addition of 1 μL of adenovirus working solution (1 × 10^8^ pfu/mL).

After 72 h of transfection, the cells were exposed to hypoxia to induce MI by culture under anoxic conditions with 93% N_2_, 2% O_2_, and 5% CO_2_. The cells cultured under normal conditions were subsequently used as control. After 24 h of culture, the subsequent experimentation was conducted.

### RT-qPCR

The cardiomyocytes were collected, and the total RNA content was extracted with TRIzol (Invitrogen™, Carlsbad, CA, USA). First strand cDNA was synthesized using the first strand cDNA synthetase according to the provided instructions of the First Strand cDNA Synthesis Kit (Takara, Tokyo, Japan). The gene expression was assessed by real-time qPCR using the SYBR Premix Ex Taq kit (Takara, Tokyo, Japan). Real time qPCR was conducted on an ABI Prism 7500 Fast Real-Time PCR system (Applied Biosystems, MA, USA). The gene expression was calculated based on the 2^-ΔΔCt^ methods with U6 and glyceraldehyde-3-phosphate dehydrogenase (GAPDH) serving as the endogenous control, and the primer sequences are listed in Table 2. As for RNA expression pattern in the mouse myocardial tissue samples, 0.1 g tissue samples were collected, and grounded at 4° C using a tissue grinder (KZ-II, Servicebio, Wuhan, Hubei, China), and the RNA content was extracted using TRIzol (Invitrogen™, Carlsbad, CA, USA) in compliance with the aforementioned procedure.

**Table 2:** Primer sequences for RT-qPCR.

**Name**	**Primer sequences**
MIAT	F: 5'-TGGAACAAGTCACGCTCGATT-3'
	R: 5'-GGTATCCCAAGGAATGAAGTCTGT-3'
miR-10a-5p	F: 5'-CGCTACCCTGTAGATCCGAATTTGTG-3'
	R: 5'-GTGCAGGGTCCGAGGT-3'
EGR2	F: 5'-GCCAAGGCCGTAGACAAAATC-3'
	R: 5'-CCACTCCGTTCATCTGGTCA-3'
GAPDH	F: 5'-AGTGCCAGCCTCGTCTCATA-3'
	R: 5'-GGTAACCAGGCGTCCGATAC-3'
U6	F: 5'-CTCGCTTCGGCAGCACA-3'
	R: 5'-AACGCTTCACGAATTTGCGT-3'

Note: miR-10a-5p, microRNA-10a-5p; MIAT, myocardial infarction-associated transcript; EGR2, early growth response gene-2; GAPDH, glyceraldehyde-3-phosphate dehydrogenase; RT-qPCR, reverse transcription quantitative polymerase chain reaction; F, forward; R, reverse.

### Western blot assay

The total protein content was extracted using the radioimmunoprecipitation assay lysis buffer (R0010, Solarbio, Beijing, China). Cell lysis was collected in an eppendorf tube, and then lysed over ice for 30 min. After centrifugation at 13,000 × g for 10 min at 4° C, the supernatant was collected, and placed over ice, and the protein concentration was determined using a bicinchoninic acid protein concentration assay kit (P0011, Beyotime, Shanghai, China). Polyacrylamide gel electrophoresis was conducted for protein separation and the protein was transferred to a 0.2 μm polyvinylidene fluoride membrane (ISEQ10100, Merck Millipore, Billerica, MA, USA) by the wet transfer. Membrane blockade was conducted with the Tris-Buffered Saline Tween-20 (TBST) (D8340, Solarbio Life Sciences Co., Ltd., Beijing, China) containing 5% skim milk powder for 1 h at ambient temperature. The primary antibodies diluted by the addition of TBST containing 1% skim milk powder were added in a drop-wise manner and incubated with the membrane at 4° C overnight, including rabbit polyclonal antibody to caspase-3 (1 : 2000, ab13847, Abcam Inc., Cambridge, UK), rabbit monoclonal antibody to Bcl-2 associated X (Bax) (1 : 2000, ab32503, Abcam), rabbit monoclonal antibody to B cell lymphoma 2 (Bcl-2) (1 : 2000, ab32124, Abcam), rabbit polyclonal antibody to EGR2 (1 : 2000, NB110-59723, Novus Biologicals, Littleton, CO, USA), and rabbit polyclonal antibody to GAPDH (1 : 4000, 10494-1-AP, Proteintech, IL, USA). On the following day, the secondary antibody horseradish peroxidase-labeled goat anti-rabbit antibody to immunoglobulin G (IgG) (1 : 5000, A0208, Beyotime, Shanghai, China) diluted by the supplementing TBST containing 1% skim milk powder was added and incubated with the experimental membrane for 1 h at ambient temperature, followed by development using a digital chemiluminometer (C-DiGit® Blot Scanner, Li-Cor, NE, USA) and analysis using the Image J software. GAPDH served as the internal reference, and the ratio of the gray value of the target band to that of the internal reference band was regarded as the relative expression of the protein.

### HE staining

The myocardial tissues of the mice were fixed in 4% paraformaldehyde (P0099, Beyotime, Shanghai, China) for 24 h, and then conventionally dehydrated with gradient alcohol of variable concentrations (70%, 80%, 90%, 95%, 100% respectively) for 5 min/time. The sections were cleared using xylene 2 times, embedded in paraffin, and sectioned systematically for a thickness of 4 μm. After baking at 60° C for 1 hour, the sections were dewaxed using xylene, dehydrated by gradient ethanol, stained with hematoxylin for 10 min, and finally differentiated using 1% hydrochloric acid alcohol for 20 s. Then, 1% ammonia water was supplemented to develop a blue color gamut for 30 s. Eosin staining was performed on the sections for 3 min, which were then dehydrated using gradient ethanol, cleared with xylene and mounted with neutral gum. Finally, the section was observed under a 40 × ordinary optical microscope (Olympus, Tokyo, Japan).

### Masson staining

Five sections after dewaxing and dehydration were stained with the Ponceau dyeing solution for 2 min, treated with 0.2% glacial acetic acid aqueous solution for 2 min, 5% phosphomolybdic acid aqueous solution for 2 min, and 0.2% glacial acetic acid aqueous solution for 2 min, and finally stained with the methyl green dye for 3 min. The color separation was conducted using 95% ethanol, followed by dehydration with ethanol, clearing with xylene, and sealing with neutral gum respectively. Under the optical microscope (100 ×), the collagen-rich fiber deformation zone was stained in blue, and the cell matrix in red, and 10 fields were randomly selected from each slice for observation. Images were documented and analyzed using the Image J image analysis software. The degree of liver fibrosis was expressed as the percentage of the fibrotic area in the entire area.

### TUNEL staining

Frozen sections of the mouse myocardial tissues were incubated in PBS containing 0.1% Triton X-100 for 5 min at ambient temperature. The subsequent steps were performed in strict accordance with the provided instructions of the TUNEL Assay Kit (C1090, Beyotime, Shanghai, China), and the TUNEL assay solution was prepared by combining the TdT enzyme and fluorescent-labeled solution at a ratio of 1 : 9. A total of 50 μL of the TUNEL assay solution was added to each sample. Samples were incubated at 37° C for 60 min in conditions devoid of light. The staining solution was discarded. Droplets of the anti-fluorescence quench were added to the surface and the samples were observed under a fluorescence microscope (Leica SR GSD, Leica, Wetzlar, Germany).

### FISH assay

FISH assay was conducted to determine the subcellular localization of MIAT in compliance with the provided instructions of the RiboTM lncRNA FISH Probe Mix (Red) (Guangzhou RiboBio Co., Ltd, Guangzhou, China), and MIAT probe was prepared according to the corresponding MIAT sequence. Briefly, the slide was placed in a 6-well plate, in which the cardiomyocytes were inoculated. After 1 day of incubation to attain 80% cell confluence, the cells were rinsed with PBS and fixed using 1 mL of 4% paraformaldehyde. After treatment with glycine and the acetylation reagent, the cells were probed with 250 μL of the pre-hybridization solution for 1 h at 42° C. After elimination of the pre-hybridization solution, 250 μL of the hybridization solution (300 ng/mL) containing the MIAT specific probe was added for overnight hybridization at 42° C. After three rinses using PBS and Tween-20 (PBST), the cells were stained using 4',6-diamidine-2-phenylindole (DAPI) diluted by PBST (1 : 800) in the 24-well plate for 5 min. After sealing with an anti-fluorescence quenching agent, the cells were observed and photographed under a fluorescence microscope (Olympus, Japan) in five randomly selected fields.

### Assessment of caspase-3 activity

The caspase-3 activity was assessed according to the provided instructions of caspase fluorescent assay kit specific for caspase-3 (Biovision, Mountain View, CA, USA). Briefly, the cardiomyocytes were lysed using the cell lysis buffer in the kit and centrifuged at 10,000 × g for 1 min at 4° C. The supernatant was collected. Using the bovine serum albumin as a standard, a protein sample of equal volume was reacted with the test solution at 37° C for 1.5 h. Finally, the absorbance value at was measured at a wavelength of 405 nm.

### Dual-luciferase reporter gene assay

The artificially synthesized EGR2 3’-untranslated region (3’UTR) was habitually introduced into the pGL3-reporter (Promega, Madison, WI, USA) using the endonuclease sites XhoI and BamH I, and the complementary sequence mutation site of the seed sequence was designed based on EGR2 WT. The target fragment was inserted into the pGL3-reporter vector using the T4 DNA ligase. The constructed luciferase reporter plasmid EGR2 WT or MUT was co-transfected into the HEK293T cells with miR-10a-5p mimic or mimic-NC, respectively. The cells were harvested and lysed 48 h after transfection, and the luciferase activity was measured using the Dual Luciferase® Reporter Assay System (E1910, Promega, Madison, WI, USA) on the Luminometer TD-20/20 detector (E5311, Promega, Madison, WI, USA). The aforementioned procedure was applicable to identify the interaction between MIAT and miR-10a-5p. The MIAT WT or MUT sequence was cloned into the pGL3-reporter vector and then co-transfected into the HEK293T cells with the miR-10a-5p mimic or mimic-NC, respectively, and then the luciferase activity was measured.

### RIP assay

The binding of MIAT or EGR2 to miR-10a-5p was detected using a RIP kit (Merck Millipore, Billerica, MA, USA). The cells were lysed with an equal volume of RIP lysis for 5 min in an ice bath, and centrifuged at 14,000 × g for 10 min at 4° C to isolate the supernatant. A portion of the cell extract was set as an input, and the remaining portion was co-precipitated with the antibody. The specific steps were as follows: 50 μL of magnetic beads were mixed with 100 μL of RIP wash buffer in each coprecipitation reaction system, after which 5 μg of antibody was added. The magnetic bead-antibody complex was re-suspended in 900 μL of RIP wash buffer, and 100 μL of the cell extract was added and incubated at 4° C overnight. The sample was placed on a magnetic base for collection of the beads-protein complex. The sample and input were respectively digested using proteinase K to extract the RNA content for subsequent PCR. The antibody used for RIP was: rabbit anti-Argonaute 2 (Ago2) (1 : 50, ab32381, Abcam Inc., Cambridge, UK).

### Flow cytometry

The cardiomyocytes were seeded in a 6-well plate, detached by supplementing a suitable amount of 0.25% trypsin (without ethylenediaminetetraacetic acid), gently re-suspended in PBS and counted. The specific steps of the protocol were conducted in reference to the provided instructions (C1062S, Beyotime, Shanghai, China). Then, 5 × 10^4^ cells were taken and centrifuged at 1000 × g for 5 min, after which the supernatant was discarded. The cell pellets were gently re-suspended by the addition of 195 μL of the Annexin V-fluorescein isothiocyanate (FITC) (C1062S, Beyotime, Shanghai, China). Next, 5 μL of Annexin-V-FITC was added, followed by addition of 10 μL of propidium iodide (PI) for incubation at ambient temperature for 10 – 20 min in conditions devoid of light. The cells were then placed on ice, and the apoptosis ratio was assessed using a flow cytometer (D3130, Novocyte™, ACEA Biosciences, CA, USA) by detecting the FITC and PI fluorescence.

### ATP content measurement

The cardiomyocytes were seeded in a 96-well plate at the concentration of 1 × 10^4^ cells per well. After treatment of the cells according to the experimental requirements, the medium of each well was replaced with fresh medium. CellTiter-Glo® working fluid (CellTiter-Glo® Luminescent Cell Viability Assay, G7570, Promega, Madison, WI, USA) was prepared according to the provided instructions and stored at -20° C. Before use, the working fluid was equilibrated to ambient temperature. Next, 100 μL of the working solution was added to each well in a 96-well plate away devoid of light, and the cells were lysed for 2 min. Then, 200 μL of the mixed liquid was transferred to an opaque 96-well plate, and 200 μL of the medium (m edium : working fluid = 1 : 1) was added as control, which was allowed to react at ambient temperature for 10 min. Detection was performed using a 96-well fluorescence detector (GloMax™, Promega, Madison, WI, USA). Then, the value of each well was normalized to the control well.

### Statistical analysis

The Statistic Package for Social Science (SPSS) 21.0 statistical software (IBM Corp, Armonk, NY, USA) was used for statistical analysis. The normal distribution and homogeneity of variance were tested accordingly. For data conforming to normal distribution and homogeneity of variance, the measurement data were summarized as mean ± standard deviation. Comparisons between two groups in the unpaired scheme were analyzed by unpaired *t* test while data comparisons among multiple groups were performed using one-way analysis of variance (ANOVA) with the Tukey’s post hoc test. A value of *p* < 0.05 was considered to be statistically significant.

### Availability of data and material

The datasets generated/analyzed during the current study are available.

## Supplementary Material

Supplementary Figure 1
