# Supplementary material for: Silencing long non-coding RNA MIAT ameliorates myocardial dysfunction induced by myocardial infarction via MIAT/miR-10a-5p/EGR2 axis
Source: Aging (Albany NY). 2021 Mar 26;13(8):11188–206. doi: 10.18632/aging.202785 (PMC8109106; doi:10.18632/aging.202785)
Supplement: Supplementary Figure 1 [file aging-13-202785-s001.pdf]

SUPPLEMENTARY FIGURE

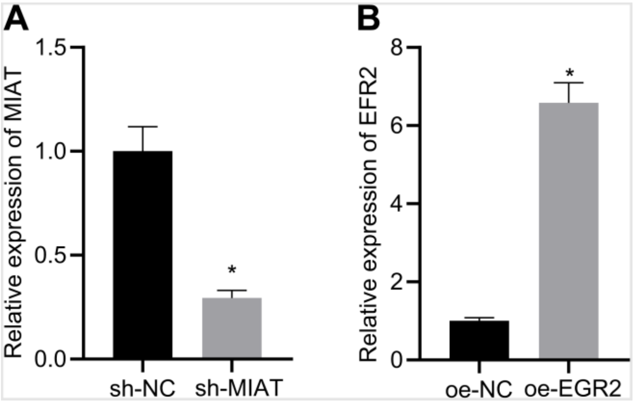

Supplementary Figure 1. MIAT is markedly silenced by treatment of sh-MIAT, and EGR2 is significantly overexpressed by treatment of oe-EGR2. (**A**) RT-qPCR determination of the efficiency of silencing MIAT. (**B**) RT-qPCR determination of the efficiency of overexpressing EGR2.
